# Supplementary material for: Cryo-electron tomography reveals coupled flavivirus replication, budding and maturation
Source: Nat Commun. 2026 Jan 20;17:828. doi: 10.1038/s41467-026-68483-4 (PMC12824359; doi:10.1038/s41467-026-68483-4)
Supplement: Supplementary file 1 — Supplementary Information [file 41467_2026_68483_MOESM1_ESM.pdf]

Supplementary Information for:

Cryo-electron tomography reveals coupled flavivirus replication, budding and maturation

This file contains:

Supplementary Figures 1-11

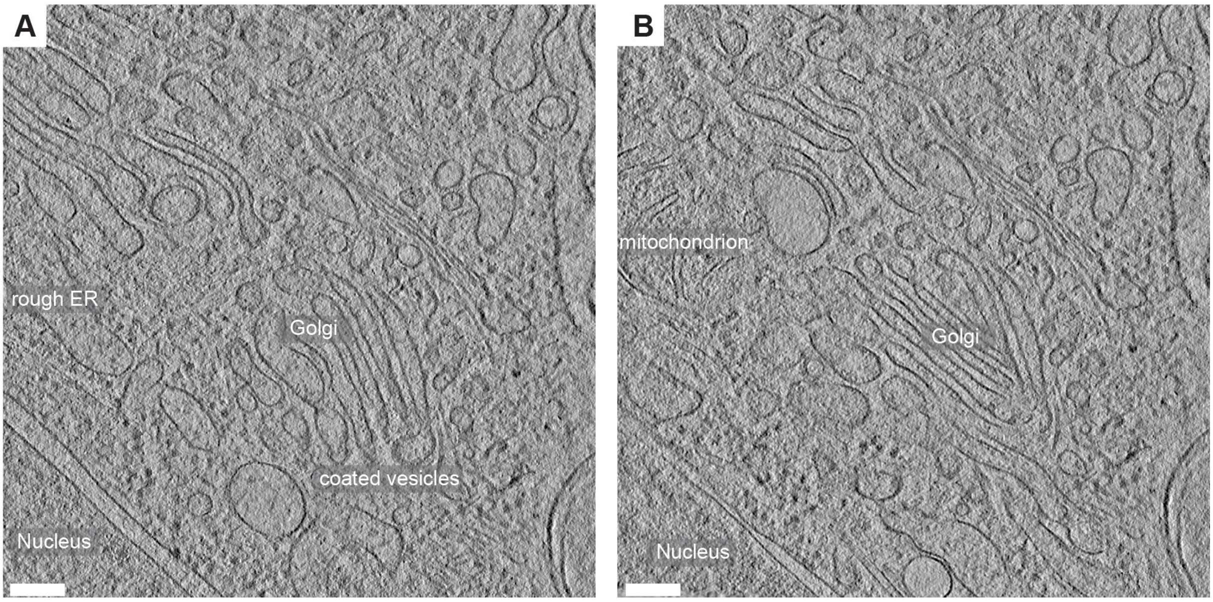

Figure S1: cryo-ET of uninfected A549 cells. (A-B) Slices from two tomograms of uninfected A549 cells reveal typical cytoplasmic features, as indicated, including a non-dilated ER and *bona fide* Golgi cisternae with typical morphology. Scale bars,100 nm.

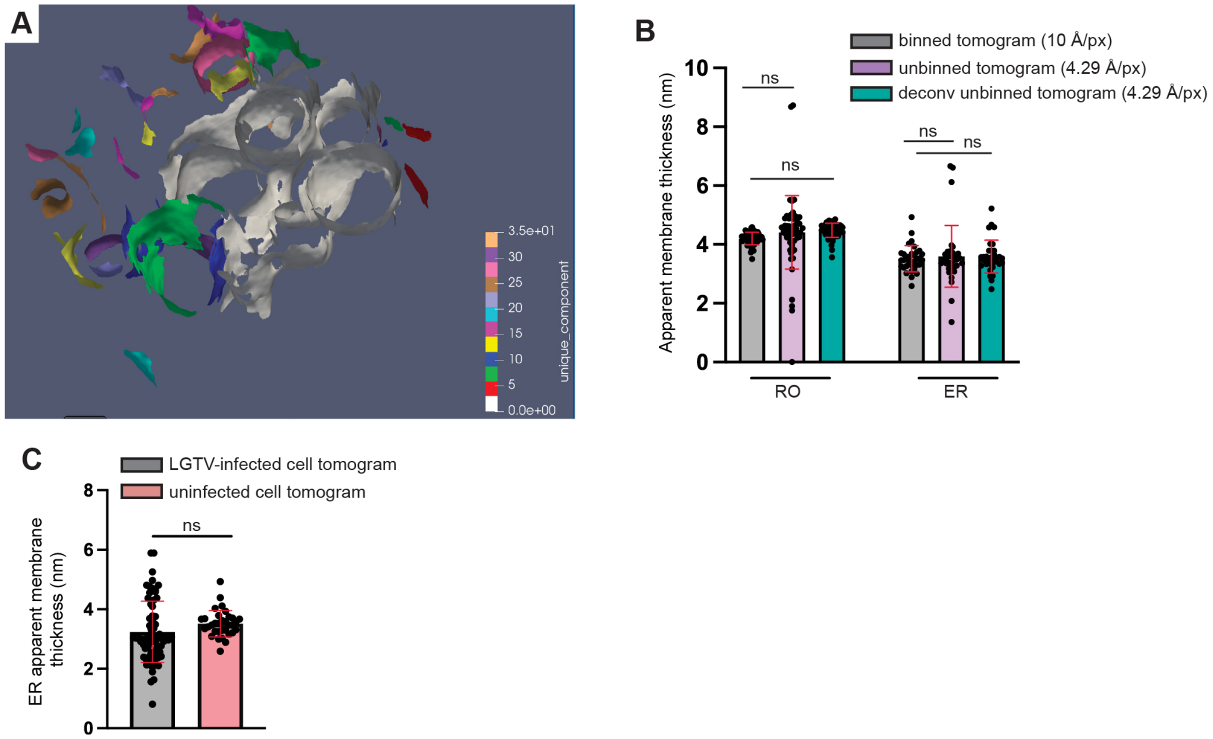

Figure S2: Membrane thickness measurements in ER and replication organelles across infection states and tomogram binning parameters. (A) ER membrane segmentation for thickness estimation. Each color represents a membrane region, within which the membrane thickness was calculated (relating to Fig. 2E). (B) Comparison of estimated membrane thickness of replication organelles and ER membranes in binned, unbinned raw, and unbinned deconvoluted data from one tomogram. Reducing binning in turn reduced signal-to-noise, leading to more observed variation without change in average; this could be corrected by applying a deconvolution filter to enhance contrast. Statistical significance was assessed by one-way ANOVA: RO (N=57, p=0.1175) and ER (N=33, p=0.8892). (C) Comparison of apparent ER membrane thickness in cryo-tomograms of LGTV-infected and uninfected cells. Statistical significance was assessed by unpaired two-tailed Student's t test (infected: N=33; uninfected: N=70; p=0.1436).

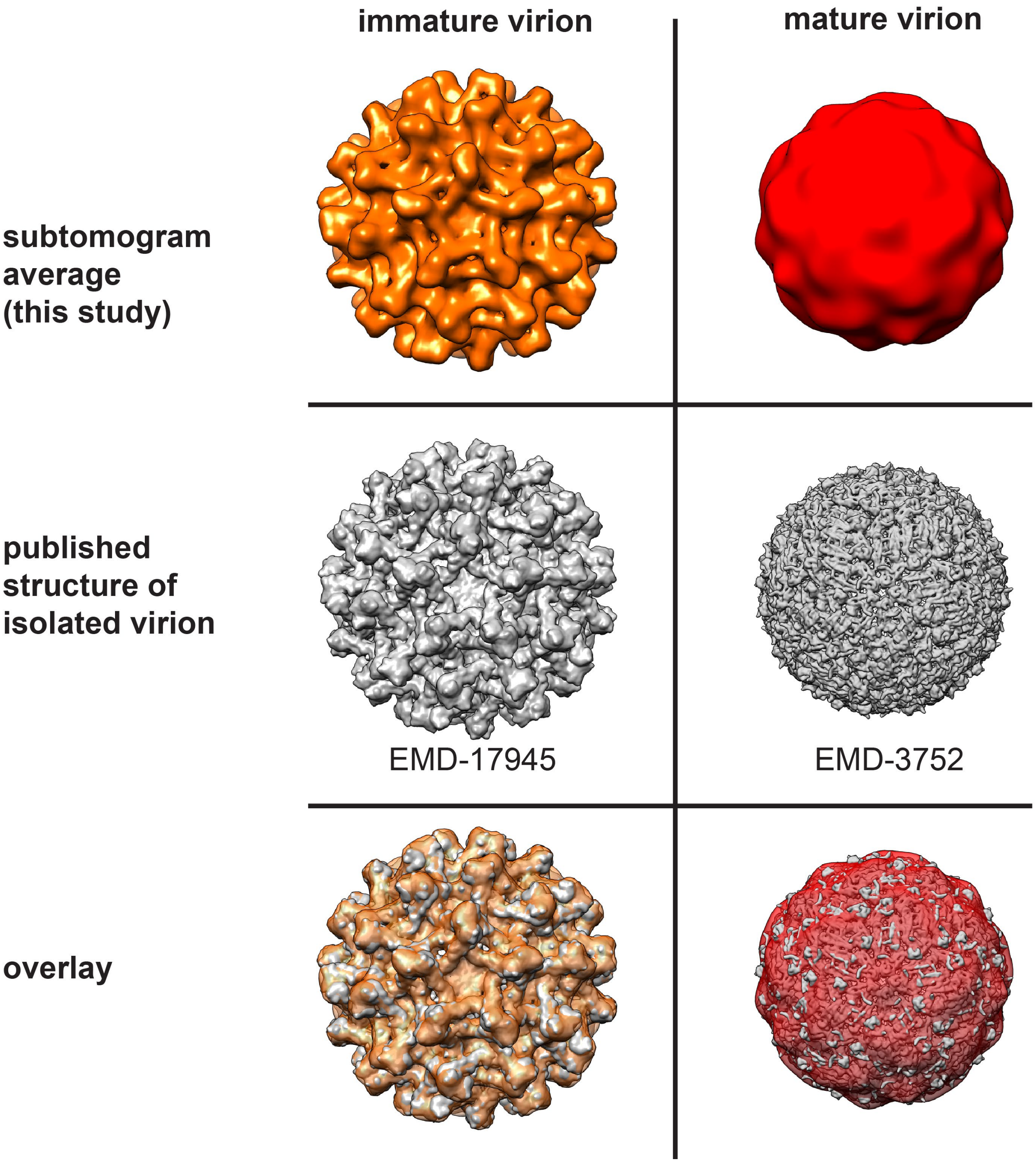

Figure S3: Comparison of cellular subtomogram averages with isolated virion structures. The cellular subtomogram averages from this study (top row) are compared to low-pass filtered published structures of immature and mature TBEV, from Fuzik *et al*[1] and Anastasina *et al*[2], respectively (mid row). The overlays (bottom row) were created using the Align to Volume command, and are shown with the subtomogram averages in semi-transparent surface representation.

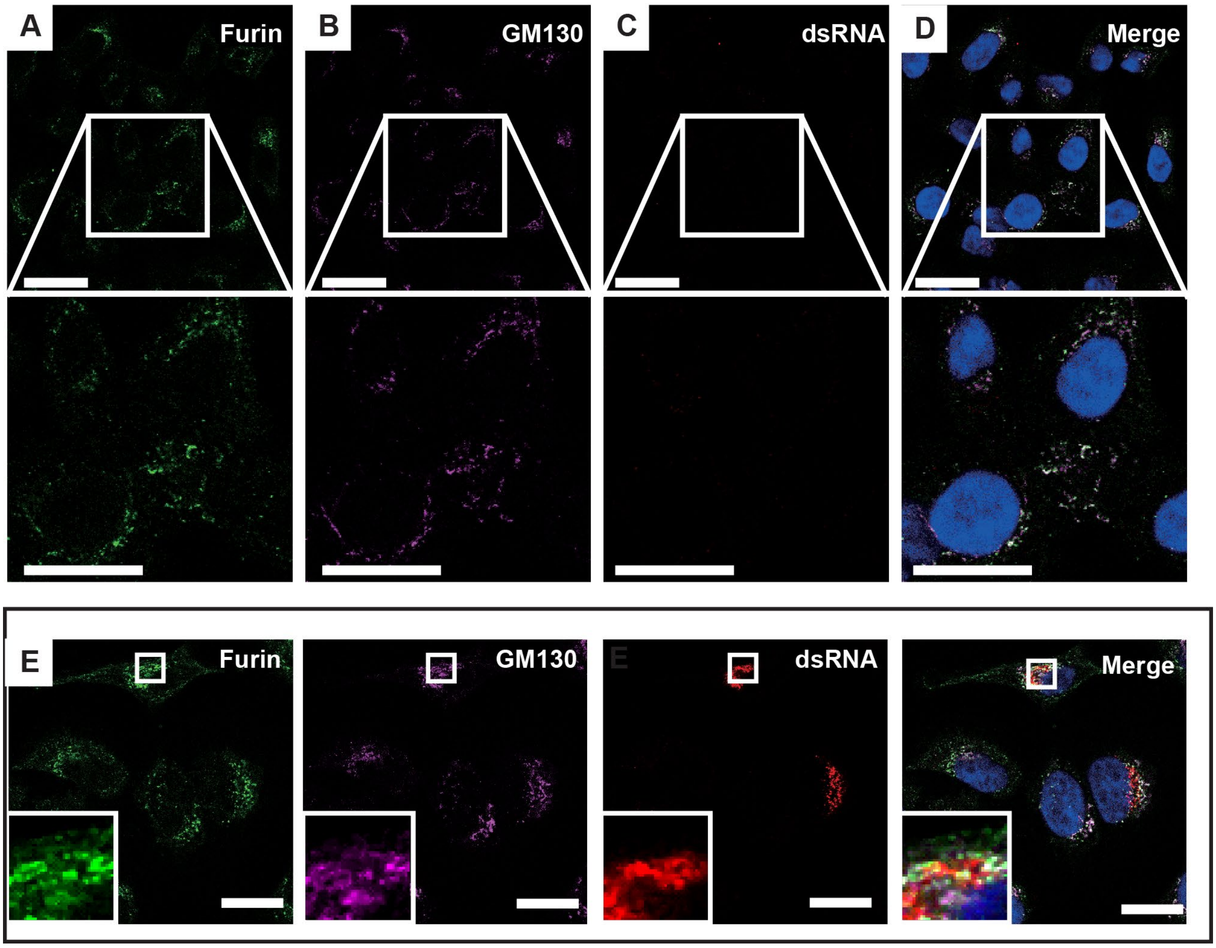

Figure S4: Furin localization in uninfected cells. (A-D) Representative immunofluorescence micrograph of uninfected cells showing furin (A) and Golgi marker GM130 (B) and their colocalization in the absence of viral infection (D). Additional channels contain dsRNA staining (C) and DAPI staining of cell nuclei (D). Scale bars, 10 µm. (E) Representative immunofluorescence micrographs of dsRNA, furin and Golgi marker GM130 in LGTV-infected cells at 24 h p.i. Rightmost panel: merge including DAPI-staining of nuclei (blue). Scale bars, 10 µm, 5 µm (E).

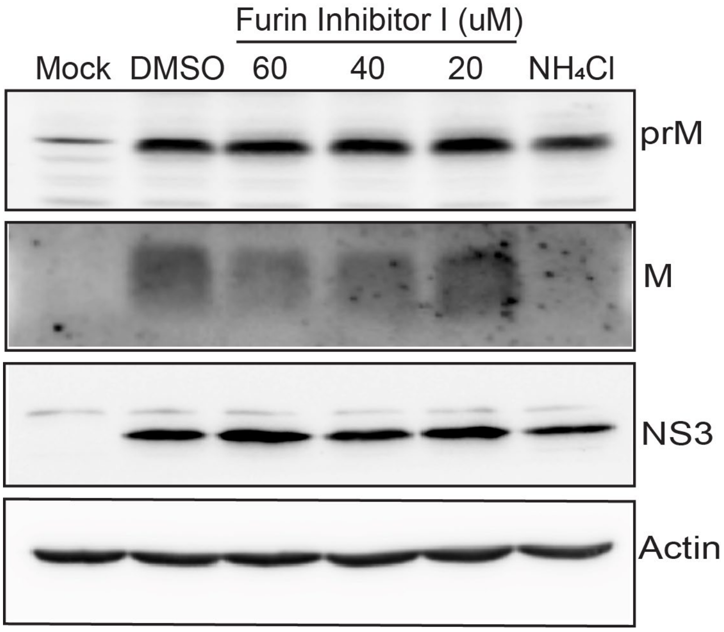

Figure S5: Western blotting of prM-to-M conversion under various conditions of furin inhibition. Representative western blots showing prM and M protein levels in mock and LGTV-infected cell lysates 24 h p.i. The infected cells were treated with DMSO, Furin Inhibitor I at indicated concentrations, or 20 mM NH_4Cl, at 15 h p.i. prM and M are detected with the same antibody on the same blot, but the M band is exposed longer due to weaker signal. Viral NS3 and cellular actin were included as infection and loading controls.

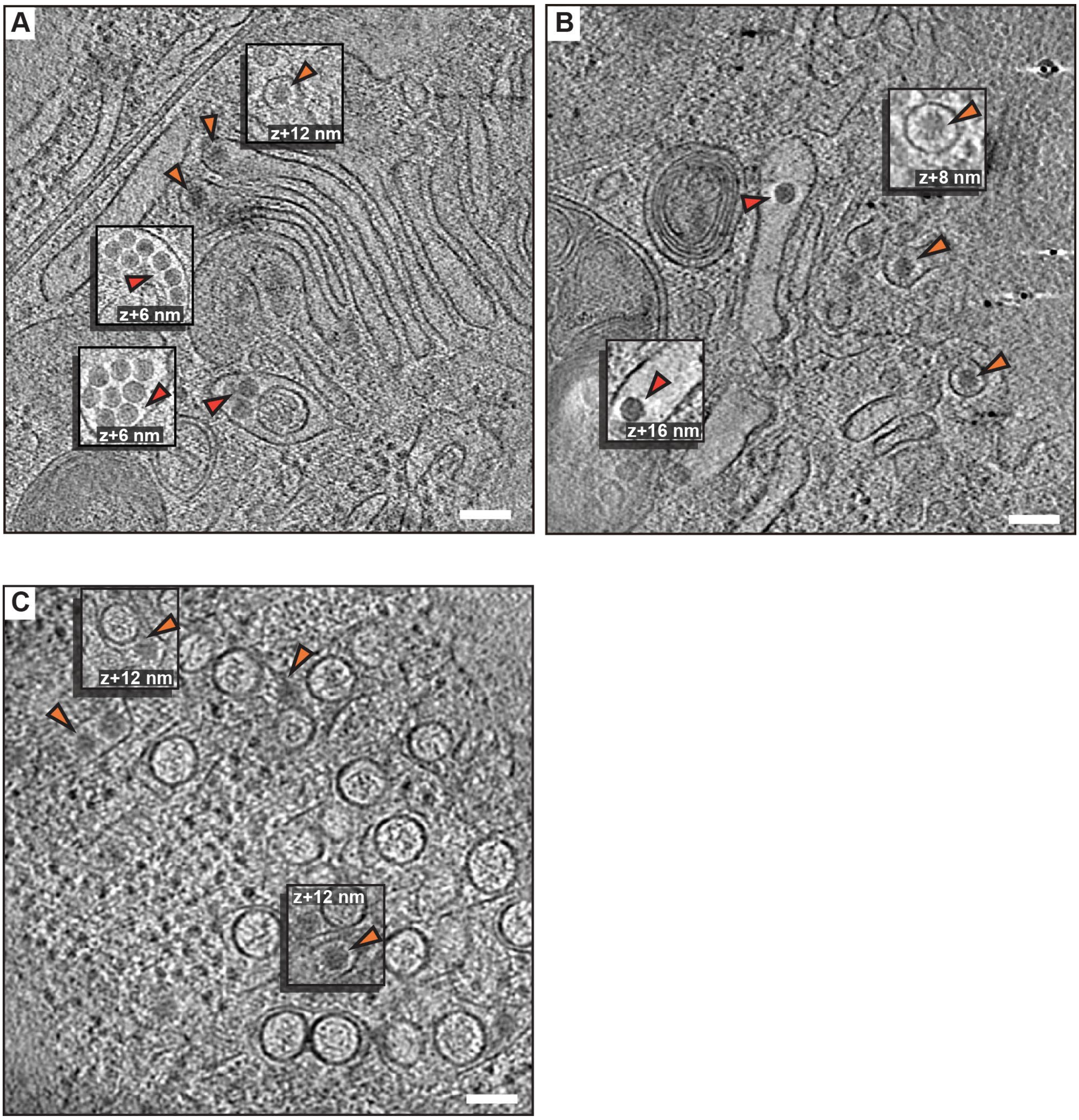

Figure S6: Cryo-ET of LGTV-infected cells under different conditions of furin inhibition. Slices through representative tomograms of LGTV-infected cells (A) untreated, (B) supplemented with 60 μM Furin Inhibitor I, and (C) supplemented with 20 mM NH_4Cl. Immature and mature virions are indicated by orange and red arrowheads, respectively. Scale bars, 50 nm.

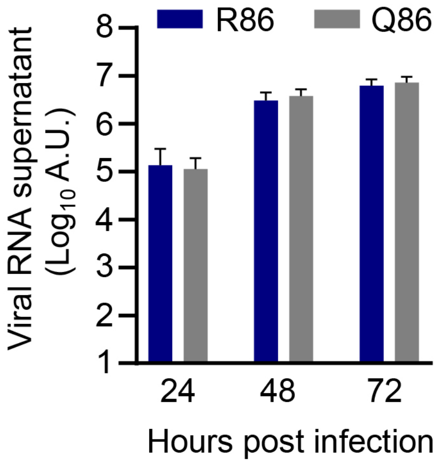

Figure S7: Replication of rLGTV$^{T:prME}$ Q86 and R86. Growth kinetics of rLGTV$^{T:prME}$ R86 and Q86 upon infection of A549 at MOI 1, quantitated as the amount of viral RNA in supernatant per qPCR.

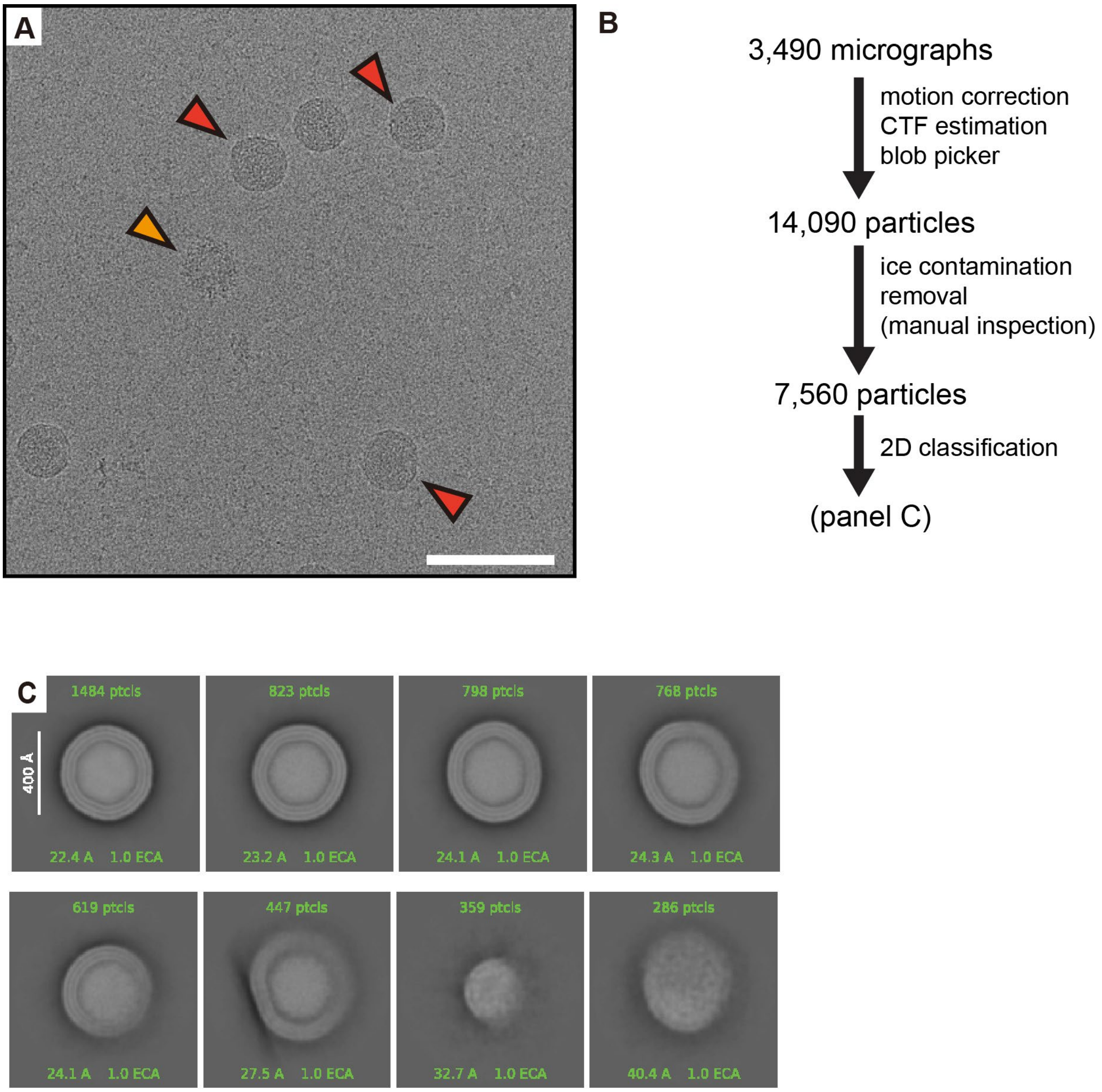

Figure S8: Cryo-EM on purified rLGTV$^{T:prME}$ Q86. (A) Representative cryo-EM micrograph of purified rLGTVT:prME Q86 showing the occurrence of a single, *bona fide* immature virion (orange arrowhead) amongst several mature-looking virions (red arrowheads). Scale bar 50 nm. (B) Data processing pipeline using CryoSPARC. (C) The most abundant 2D class averages, representing 74% of the 7,560 virions, showing primarily mature-morphology particles.

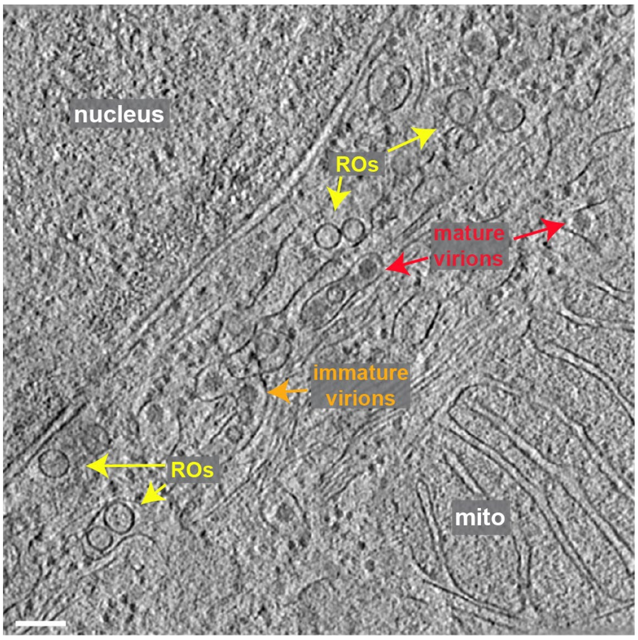

Figure S9: Cryo-ET of a cell infected with rLGTV$^{T:prME}$ R86. Slice through a representative tomogram acquired at 24 h p.i. showing various cytoplasmic and virus-related features, as indicated. Scale bar 100 nm.

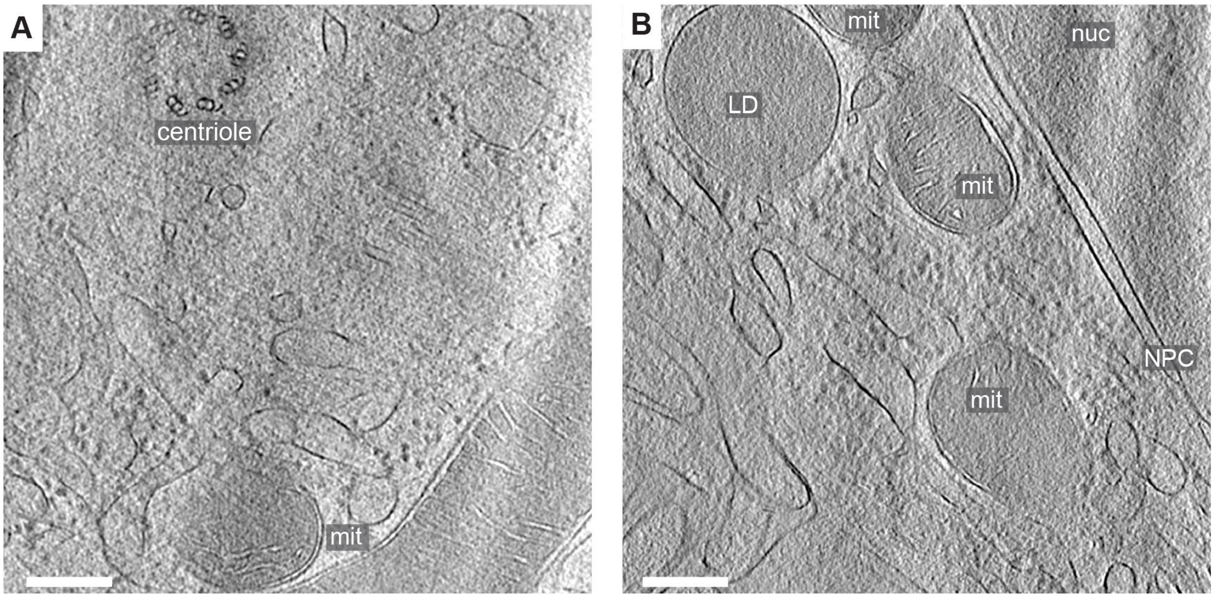

Figure S10: Features unrelated to infection in cryo-electron tomograms of *ex vivo* brain tissue. (A-B) Slices from two tomograms of high-pressure frozen choroid plexus from LGTV-infected *Ifnar*$^{-/-}$ mice. The features indicated are a centriole, several mitochondria (mit), a lipid droplet (LD), the peripheral area of a nucleus (nuc) and the nuclear envelope including one nuclear pore complex (NPC). Scale bar, 100 nm.

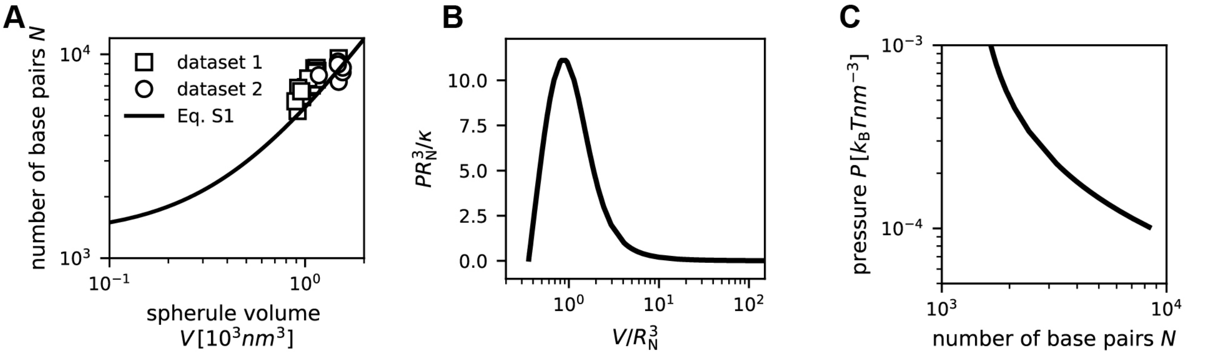

Figure S11. Pressure exerted by an RNA strand. (A) Relation between number of RNA base pairs and RO volume. The data is reproduced from Laurent *et al*[3]. We note that in Laurent *et al*[3] the RNA length is shown, while here the number of base pairs is shown, assuming an interbasepair distance of 2.56 Å. (B) Relation between the scaled pressure and the scaled volume. The details of the underlying model are presented in Laurent *et al*[3]. (C) Relation between number of RNA base pairs N and pressure P, where we use the results from (A-B) to convert the RO volume into number of RNA base pairs.

References for supplementary figures

1 Fuzik, T. *et al.* Structure of tick-borne encephalitis virus and its neutralization by a monoclonal antibody. *Nature communications* **9**, 436 (2018). https://doi.org/10.1038/s41467-018-02882-0

2 Anastasina, M. *et al.* The structure of immature tick-borne encephalitis virus supports the collapse model of flavivirus maturation. *Sci Adv* **10**, eadl1888 (2024). https://doi.org/10.1126/sciadv.adl1888

3 Laurent, T. *et al.* Architecture of the chikungunya virus replication organelle. *eLife* **11** (2022). https://doi.org/10.7554/eLife.83042
