# Supplementary material for: Cryo-electron tomography reveals coupled flavivirus replication, budding and maturation
Source: Nat Commun. 2026 Jan 20;17:828. doi: 10.1038/s41467-026-68483-4 (PMC12824359; doi:10.1038/s41467-026-68483-4)
Supplement: Supplementary file 2 — Description Of Additional Supplementary File [file 41467_2026_68483_MOESM2_ESM.pdf]

Description of Additional supplementary files

Title: Supplementary Movie 1.

Description: Tomographic volume corresponding to the slice shown in Fig. 1A.

Title: Supplementary Movie 2.

Description: The segmentation shown in Fig. 1D.

Title: Supplementary Movie 3.

Description: Tomographic volume corresponding to the slice shown in Fig. 3A.

Title: Supplementary Movie 4.

Description: The segmentation shown in Fig. 3B.

Title: Supplementary Movie 5.

Description: Tomographic volume corresponding to the slice shown in Fig. 4J (rotated clockwise 90 degrees with respect to the figure).

Title: Supplementary Movie 6.

Description: The segmentation shown in Fig. 4K (rotated clockwise 90 degrees with respect to the figure).

Title: Supplementary Movie 7.

Description: Tomographic volume corresponding to the slices shown in Fig. 5F-G.

Title: Supplementary Movie 8.

Description: The segmentation shown in Fig. 5H.

Title: Supplementary Movie 9.

Description: Tomographic volume corresponding to the slice shown in Fig.6B.
