# Supplementary material for: Cryo-electron tomography reveals coupled flavivirus replication, budding and maturation
Source: Nat Commun. 2026 Jan 20;17:828. doi: 10.1038/s41467-026-68483-4 (PMC12824359; doi:10.1038/s41467-026-68483-4)
Supplement: Supplementary file 12 — Reporting summary [file 41467_2026_68483_MOESM12_ESM.pdf]

nature portfolio

Corresponding author(s): Selma Dahmane, Ben Barad, Anna Överby, Lars-Anders Carlson (LAC filled out this document)

Last updated by author(s): Jan 1, 2026

Reporting Summary

Nature Portfolio wishes to improve the reproducibility of the work that we publish. This form provides structure for consistency and transparency in reporting. For further information on Nature Portfolio policies, see our Editorial Policies and the Editorial Policy Checklist.

Statistics

For all statistical analyses, confirm that the following items are present in the figure legend, table legend, main text, or Methods section.

n/a	Confirmed	
☐	☒	The exact sample size (*n*) for each experimental group/condition, given as a discrete number and unit of measurement
☐	☒	A statement on whether measurements were taken from distinct samples or whether the same sample was measured repeatedly
☐	☒	The statistical test(s) used AND whether they are one- or two-sided<br>*Only common tests should be described solely by name; describe more complex techniques in the Methods section.*
☒	☐	A description of all covariates tested
☒	☐	A description of any assumptions or corrections, such as tests of normality and adjustment for multiple comparisons
☐	☒	A full description of the statistical parameters including central tendency (e.g. means) or other basic estimates (e.g. regression coefficient) AND variation (e.g. standard deviation) or associated estimates of uncertainty (e.g. confidence intervals)
☐	☒	For null hypothesis testing, the test statistic (e.g. *F*, *t*, *r*) with confidence intervals, effect sizes, degrees of freedom and *P* value noted<br>*Give P values as exact values whenever suitable.*
☒	☐	For Bayesian analysis, information on the choice of priors and Markov chain Monte Carlo settings
☒	☐	For hierarchical and complex designs, identification of the appropriate level for tests and full reporting of outcomes
☒	☐	Estimates of effect sizes (e.g. Cohen's *d*, Pearson's *r*), indicating how they were calculated

Our web collection on statistics for biologists contains articles on many of the points above.

Software and code

Policy information about availability of computer code

Data collection	SerialEM 3.8; Leica LAS X
Data analysis	MotionCor2 1.3.1; CTFFIND4 4.1.8; IMOD 4.10; cryoCARE 0.3; IsoNet 0.2; Amira 2023.1.1; MemBrain-seg v0.0.5;<br>UCSF Chimera 1.15; WARP 1.0.9; Dynamo 1.1.509; Prism 10<br>surface morphometrics https://github.com/GrotjahnLab/surface_morphometrics

For manuscripts utilizing custom algorithms or software that are central to the research but not yet described in published literature, software must be made available to editors and reviewers. We strongly encourage code deposition in a community repository (e.g. GitHub). See the Nature Portfolio guidelines for submitting code & software for further information.

Data

Policy information about availability of data

All manuscripts must include a data availability statement. This statement should provide the following information, where applicable:

- Accession codes, unique identifiers, or web links for publicly available datasets
- A description of any restrictions on data availability
- For clinical datasets or third party data, please ensure that the statement adheres to our policy

The cellular subtomogram averages of immature and mature Langat virus are deposited at the Electron Microscopy Data Bank with accession codes EMD-51640 and EMD-51642, respectively.

Research involving human participants, their data, or biological material

Policy information about studies with human participants or human data. See also policy information about sex, gender (identity/presentation), and sexual orientation and race, ethnicity and racism.

Reporting on sex and gender	N/A
Reporting on race, ethnicity, or other socially relevant groupings	N/A
Population characteristics	N/A
Recruitment	N/A
Ethics oversight	N/A

Note that full information on the approval of the study protocol must also be provided in the manuscript.

Field-specific reporting

Please select the one below that is the best fit for your research. If you are not sure, read the appropriate sections before making your selection.

☒ Life sciences ☐ Behavioural & social sciences ☐ Ecological, evolutionary & environmental sciences

For a reference copy of the document with all sections, see nature.com/documents/nr-reporting-summary-flat.pdf

Life sciences study design

All studies must disclose on these points even when the disclosure is negative.

Sample size	No sample size calculation was performed.
Data exclusions	Cryo-electron tomography data were excluded based solely on technical data quality parameters.
Replication	There were no unsuccessful attempts at replication.
Randomization	N/A
Blinding	There was no blinding. It is not relevant to this type of study in our opinion.

Reporting for specific materials, systems and methods

We require information from authors about some types of materials, experimental systems and methods used in many studies. Here, indicate whether each material, system or method listed is relevant to your study. If you are not sure if a list item applies to your research, read the appropriate section before selecting a response.

Materials & experimental systems

n/a	Involved in the study
☐	☒ Antibodies
☐	☒ Eukaryotic cell lines
☒	☐ Palaeontology and archaeology
☐	☒ Animals and other organisms
☒	☐ Clinical data
☒	☐ Dual use research of concern
☒	☐ Plants

Methods

n/a	Involved in the study
☒	☐ ChIP-seq
☒	☐ Flow cytometry
☒	☐ MRI-based neuroimaging

Antibodies

Antibodies used	Anti-furin: goat polyclonal, dilution 1:50, Invitrogen; Anti-GM130: mouse monoclonal clone 35/GM130, BD Transduction; Anti-dsRNA: mouse monoclonal clone J2, Scicons, Nordic MUbio; conjugated to allophycocyanin, Abcam; donkey anti-goat Alexa Fluor 488, Invitrogen; goat anti-mouse Alexa Fluor 568, Invitrogen
Validation	Antibodies validated by manufacturer.

Eukaryotic cell lines

Policy information about cell lines and Sex and Gender in Research

Cell line source(s)	A549: human lung epithelial cell line
Authentication	the cell line was not independently authenticated
Mycoplasma contamination	The cell line was tested negative for mycoplasma by PCR.
Commonly misidentified lines (See ICLAC register)	*Name any commonly misidentified cell lines used in the study and provide a rationale for their use.*

Animals and other research organisms

Policy information about studies involving animals; ARRIVE guidelines recommended for reporting animal research, and Sex and Gender in Research

Laboratory animals	Mavs-/- mice in C57BL/6 background (kind gift of Nelson O Gekara, Umeå University); Ifnar–/– mice
Wild animals	No wild animals
Reporting on sex	This information was not collected
Field-collected samples	No samples were collected from the field.
Ethics oversight	All animal experiments were approved by the regional Animal Research Ethics Committee of Northern Norrland and the Swedish Board of Agriculture, ethical permit A9-2018, A41-2019

Note that full information on the approval of the study protocol must also be provided in the manuscript.

Plants

Seed stocks	N/A
Novel plant genotypes	N/A
Authentication	N/A
